# Biosensors for the Detection of Food Pathogens

**DOI:** 10.3390/foods3030511

**Published:** 2014-09-02

**Authors:** Palmiro Poltronieri, Valeria Mezzolla, Elisabetta Primiceri, Giuseppe Maruccio

**Affiliations:** 1 Institute of Sciences of Food Productions, National Research Council, ISPA-CNR, Via Lecce-Monteroni, 73100 Lecce, Italy; E-Mail: valeria.mezzolla@gmail.com; 2 NNL, Institute of Nanoscience-CNR, Via per Arnesano, I-73100 Lecce, Italy; E-Mails: elisabetta.primiceri@unisalento.it (E.P.); giuseppe.maruccio@unisalento.it (G.M.); 3 Department of Mathematics and Physics “Ennio De Giorgi”, University of Salento, Via per Arnesano, I-73100 Lecce, Italy

**Keywords:** food pathogens, rapid identification, molecular recognition, detection methods, labelling, antibody chips, lab-on-a-chip

## Abstract

Food pathogens frequently cause foodborne diseases. There is a need to rapidly identify the source of the bacteria in order to contain their spread and epidemics. A pre-enrichment culture or a direct culture on agar plate are standard microbiological methods. In this review, we present an update on alternative molecular methods to nucleic acid-based detection for species identification. Biosensor-based methods rely on the recognition of antigen targets or receptors by antibodies, aptamers or high-affinity ligands. The captured antigens may be then directly or indirectly detected through an antibody or high-affinity and high-specificity recognition molecule. Various different detection methods are discussed, from label-free sensors and immunosensors to fluorescence-based ones. Each method shows advantages and disadvantages in terms of equipment, sensitivity, simplicity and cost-effectiveness. Finally, lab-on-a-chip (LOC) devices are introduced briefly, with the potential to be fast, sensitive and useful for on-site bacteria detection in food processing laboratories to check potential contamination by sample monitoring combined with a rapid pre-enrichment step.

## 1. Introduction

Numerous cases of foodborne illness among humans are caused by pathogens delivered with foods. In addition to human health risks, microbial contamination can result in food spoilage. The most common foodborne infections are caused by *Campylobacter* spp*.*, *Salmonella* spp*.* and* Escherichia coli* O157:H7. Other bacterial species, such as *Listeria monocytogenes*,* Bacillus cereus*, *Bacillus anthracis*, the enterohemorrhagic strains of *Escherichia coli* O103:H25, O26, O111, O115, O128, O145 and the recently isolated O104:H4 harboring an antibiotic resistance plasmid, *Staphylococcus aureus* strains coding for thermostable enterotoxins, *Clostridium botulinum*, *Clostridium perfringens*, *Yersinia enterocolitica*, *Vibrio* spp., *Aeromonas* spp., *Shigella* spp. and *Mycobacterium bovis*, viruses and protozoa are transmitted through food consumption (source: Center for Disease Control, USA) [1].

Raw meat, milk, seeds and vegetables may be the source of bacteria, which are transferred through cross-contamination during food preparation, reaching the food supply chain. Certain types of foods (milk, cream, meat) are required to be free of pathogens, such as *L. monocytogenes* and *S. aureus*. The threshold for the acceptance of a pathogen depends on its ability to grow at refrigerated conditions, at which psychrotrophic bacteria, such as *L. monocytogenes*, are able to proliferate. On the contrary, the presence of *Legionella pneumophila* in water pipelines in hotels and thermal baths is considered a health threat when the bacteria density reaches several thousands of cells in one liter of water [2].

The standard microbiology method in food pathogen detection is colony counting on an agar plate, a procedure that requires several days for revealing the presence of a pathogen. This review will focus on methods for the detection of bacteria in food samples incubated in a pre-enrichment broth for times shorter than the standard 24–48 h, in order to individuate the bacteria, even if the exponential growth phase has not reached its maximum peak. A concentration step is required to collect bacteria from larger volumes into a small area, such as through filtration or using immuno-magnetic nanoparticles or beads loaded with species-specific antibodies or epitope binding proteins. Many publications have exploited the immunomagnetic separation of bacteria from a solution or food resuspension. Nowadays, the Pathatrix Auto System (Life Technologies, Carlsbad, CA, USA) has been validated and approved by AOAC for *Salmonella* spp. and *Listeria* spp. detection. Using these concentrators, bacteria from an enriched culture of 15 mL incubated for 15 h can be processed through DNA purification and amplification or immunodetection [3].

The highest sensitivity in species identification of bacteria has been achieved with molecular methods based both on polymerase chain reaction (real-time PCR, digital PCR) and isothermal amplification methods, like rolling circle amplification (RCA), recombinase polymerase amplification (RPA) and loop-mediated isothermal amplification (LAMP). In addition to the quantitative evaluation of the amplified DNA by quantitative real-time PCR, other methods have been developed, which are based on the hybridization of target DNA with highly selective probes bound to a surface. The detection limit of DNA methods range between 10 to 100 colony forming units/mL of sample [4,5].

While several methods have been applied for species identification after bacteria have reached the exponential phase in culture broth, there is a need to overcome remaining bottlenecks in the microbiological analysis of foods. For example, the food matrix is a constraint for the elution of bacteria and liberates PCR inhibitors that may affect the subsequent analyses. In addition, the bacteria are stressed and require longer times to grow. 

Two identification methods can be applied in food safety based on ISO certification: plating and counting on selective agar and real-time PCR with dedicated DNA extraction kits (Bio-Rad) applied to bacteria from enrichment broths at 24 or 48 h of incubation. This molecular method has proven to be sensitive enough to detect 10 to 100 bacteria (from volumes up to 50 mL when combined with immunomagnetic separation) [4,5,6].

Methods, such as lateral flow-based dip-sticks with species-specific antibodies, and generally all immunosensors are not sensitive enough, and for this reason, they are used for species identification in enrichment broths at 24–48 h of incubation.

Nowadays, the developed immunoassays are based upon bacterial species-specific antibodies, aptamers [7,8] and immuno-recognition of bacterial antigens (such as bacteriophage tailspike protein) [9]. These methods require standard conditions for the optimum binding of proteins or other highly-specific affinity compounds on: beads (Luminex, Austin, TX, USA), glass slides, gold surfaces, microplates and membranes suitable for chromatographic separation of antigen-antibody complexes, combined with dipsticks, microfluidic channels on paper (μPADs) [10] or lateral flow immuno-assays (LFIA), in which the capture antibody is conjugated with a detection molecule exploiting colorimetric methods, chemiluminescence or gold nanoparticles [11]. We found that the sensitivity of this last method applied in *Salmonella* spp. detection was approximately 10^5^ CFU/mL, thus making it unsuitable for detection in pre-enrichment broth at early stages of growth (18–24 h). 

Presently many biosensor-based methods are still labor-intensive, expensive, and not easily implementable for in-field applications. 

## 2. Detection Methods in Label-Free Sensors and Immunosensors 

An ideal biosensor should detect target molecules directly without the use of labelled ligands or multiple washing steps. There are several bottlenecks to be solved in order to perform an efficient analysis. The first is the use of proper surfaces. The second is to efficiently and uniformly print the capture probe (antibody, binding proteins, aptamers) and to have a constant probe density in order to obtain the detection of targets with high reproducibility. The third is to obtain a high sensibility even at a low concentration of the targets. A number of methods are used for the rapid detection of biomolecules in solution. These include immunoassays, chromatographic methods, magnetic and biological biosensor methods (using screen-printed biosensors with immobilized enzymes), DNA biosensors and antibody-based detection methods.

### 2.1. Surface Plasmon Resonance (SPR)

SPR biosensors have been used in the direct and indirect detection of pathogenic microorganisms [12]. The technique was largely studied in label-free immunosensors for the detection of bacteria [13,14,15]. SPR can be used for the setup of immunosensors applied to the detection of food pathogens in enrichment broth, in liquids or in food dilutions. 

The SPR technique for biosensing allows real-time monitoring of chemical and bio-chemical interactions occurring at the interface between a thin gold film and a dielectric interface or transparent material, such as the liquid analyte. SPR principles have been deeply described and discussed in other reviews [16]. SPR is an optical technique that uses the evanescent wave phenomenon to measure changes in refractive index very close to the sensor surface. The evanescent wave produced by an incident, monochromatic light beam is able to interact with free electrons (plasmons) in the metal film at a special angle (α) of incident light (SPR angle). The angular position is highly sensitive to any change at the metal-dielectric interface. Many real-time monitoring SPR methods exploit the prism-based Kretschmann configuration that uses the excitation of a surface-bound electromagnetic wave from the metal side. The occurrence of a binding event between the investigated antigen and antibody can be monitored by following the shift of the angular minimum toward higher angles or the variation in reflectance at a fixed angle (corresponding to the maximum slope of the curve). Thus, it is possible to record the SPR reflectance curve at different angles of incidence of light. In a report, a *L. pneumophila* cell suspension at a concentration of 10,000 CFU/mL was detected using an SPR immunosensor provided with a miniaturized microfluidic system [2]. 

#### 2.1.1. SPR Imaging

SPR microscopy is suitable for the evaluation of end-point quantification. In grating-coupled SPR, macroscopically-thick metal films are deposited on inexpensive and disposable grating. When the wave is excited from the transparent material side, in the Otto-configuration, the sample is placed on a topographically-modified surface, such as a grating or a grid printed by spot deposition in a microarray format [17]. Such a method does not require a prism. The surface, even if not in gold, does not need to be uniformly thick. Grating-coupled surface plasmon resonance imaging (GCSPRI) has been applied to multiplexed detection of microbes, toxins and viruses. GCSPRI instrument showed the ability to simultaneously measure binding at over 1000 unique, discrete regions of interest (ROIs) by utilizing a compact microarray of antibodies or other specific capture molecules immobilized on the sensor chip.

#### 2.1.2. Enhancement of Sensitivity by Combining SPR with A Labelling or Capturing Method

Standard SPR detection requires usually high concentrations of the screened target antigens. This may lead to non-specific binding and a limit of detection of bacteria in the range of 10^5^–10^6^ CFU/mL. For this reason, improvement of the SPR detection limit has been achieved with an additional labelling step.

To enhance the signal from bacteria bound to the antibodies on the gold surface [18], antibody-functionalized gold nanoparticles (immuno-AuNP) were allowed to flow into the SPR cell, producing an increase in the reflectance units already at an *L. monocytogenes* concentration of 1000 CFU/mL [4]. The sensitivity was significantly enhanced, based on a mass effect.

A new, indirect method capable of enhancing SPR sensitivity is label-enhanced surface plasmon fluorescence. An alternative to a fluorophore, to avoid quenching problems due to the gold surface, a more efficient method based on the absorbance of light is quantitative label-enhanced SPR [19]. Label-enhanced SPR can be run simultaneously and in parallel to label-free SPR. The specificity is very high, and the influence of environment factors, such as temperature and buffer composition variations, is reduced.

In the subtractive inhibition assay, *E. coli* O157:H7 cells and goat polyclonal antibodies for* E. coli* O157:H7 were incubated for a short amount of time, and then, the *E. coli* O157:H7 cells, to which antibodies were bound, were removed by a stepwise centrifugation process. The remaining free unbound antibodies were detected through an interaction with rabbit anti-goat IgG polyclonal antibodies immobilized on the sensor chip using a BIAcore 3000 biosensor. The results showed that the signal was inversely correlated with the concentration of *E. coli* O157:H7 cells in a range from 3.0 × 10^4^ to 3.0 × 10^8^ CFU/mL. Compared to direct SPR, the detection limit of the subtractive inhibition assay method was reduced by one order of magnitude [20].

Lectins have been used to capture the bacteria on the gold surface, in the development of surface plasmon resonance biosensors. In this prototype, the adhesion of bacteria depends on the affinity between specific lectins and surface carbohydrates [21].

### 2.2. Electrochemical Impedance Spectroscopy (EIS)

Electrochemical impedance spectroscopy (EIS) has been extensively applied as a method of choice for biosensors [22], combined with disposable, screen-printed chips [23]. Each electrode can be disposed after use in order to prevent carry-over contamination. Due to its high sensitivity and easy setup, EIS has been extensively applied for biosensor fabrication, particularly in the field of microbiology. Since the 1990s, impedance methods have been used for bacterial identification. These methods record the changes in the sensor electrical impedance induced by bacterial metabolism and cell growth as a result of the release of ionic metabolites from the living cells (carbon dioxide and organic acids produced by catabolism and ion exchange through the cell membrane). One application of this technique has been applied to differentiate between viable and dead cells [24,25]. The method has been used also by Yang and coworkers [26,27], who applied a three-electrode configuration for the impedimetric detection of viable *S. typhimurium*. Since no changes happen at the electrodes surface, the system can be described by a simplified equivalent circuit composed of a resistor (R_s_) associated with the solution between the two electrodes and a double-layer capacitor for each electrode (C_dl_). These two components determine the total (frequency-dependent) impedance. At high frequencies, the medium resistance is essentially the main contributor to the total impedance; whereas at low frequencies, the double-layer capacitance dominates. It was shown that both the capacitance and resistance measurements can be used for the enumeration of bacteria [26].

In the past few years, advances in microfabrication technologies allowed a miniaturization of the impedimetric biosensors into a chip format offering new opportunities in the field of bacterial detection. Several advantages are provided, since dedicated chips can be mass produced at a low cost, so each electrode can be disposed of after use, avoiding cross-contamination [26]. In this respect, interdigitated microelectrodes (IME) were used for the quantitative detection of *S. typhimurium* in pure culture and milk samples by means of impedance measurements [27]. Specifically, low frequencies (<100 Hz) were suitable to monitor the impedance changes associated with bacterial growth, and a significant change in impedance at 10 Hz was recorded because of the increase in the double-layer capacitance, while a simple measurement of the resistance is not sufficient, since bacteria cells attached to the electrode surface cause an increase in resistance, while a decrease in medium resistance limits the evaluation of total impedance. The detection time of the IME impedance sensor for *S. typhimurium* at a given initial cell number was shorter than that by the classic impedimetry and was close to that of electrochemical methods. A detection limit as low as 0.5 CFU/mL with a detection time of more than 10 h was reported [27].

In addition, microfabricated systems allow for the functionalization of the electrode surface with molecules able to capture specific bacterial cells. In this case, the equivalent circuit consists of the ohmic resistance of the electrolytic solution (R_s_), a double-layer capacitance (C_dl_), an interfacial electron-transfer resistance (R_et_) and a Warburg impedance (Z_w_), which is associated with the diffusion of the redox probe. Among them, C_dl_ and R_et _depend on the electrical properties of the solution/electrode interface and can be used to monitor the attachment of molecules [28] and cells [29]. As a result of specific biorecognition reactions, this method allows the detection of a specific type of bacteria or different bacteria present at the same time, even in complex matrices, thanks to a strong reduction of unspecific contributions to the signal. Moreover, the time needed for the analysis can be strongly reduced (less than 2 h) compared with growth-based impedance methods (which requires at least 24 h). For example, in 2002, Ruan *et al.* [30] developed an impedance biosensor for the detection of *Escherichia coli* O157:H7 based on the immobilization of bacteria to electrodes functionalized with specific antibodies. A secondary antibody conjugated with alkaline phosphatase as the labeled enzyme was used to amplify the signals. These biosensors were able to detect the target bacteria with a detection limit of 6 × 10^3^ cells/mL. Yang *et al*. [31] were able to avoid the use of secondary labelled antibodies and develop label-free immunosensors for the detection of the same bacterial strain (*E. coli* O157:H7) by exploiting the better sensitivity of indium tin oxide (ITO) interdigitated electrodes, due to the compatibility in terms of size between the electrodes and bacterial cells. In fact, when bacterial cells are recognized by the specific antibodies immobilized on the electrodes, they are attached to the surface and induce an increase in the electron transfer resistance, measurable even without any additional amplification step. More recently, Radhakrishnan and colleagues [32] were able to detect *L. monocytogenes* by electrochemical impedance spectroscopy with very high sensitivity using a mouse monoclonal antibody immobilized onto a gold electrode. They reported detection limits of 5 CFU/mL and 4 CFU/mL for ideal solutions and filtered tomato extract, respectively.

To increase the sensitivity of the assays, the use of innovative materials for electrode fabrication was also investigated, and in this respect, nanomaterials, such as nanoparticles, nanowires, nanotubes and graphene, seem to be very promising. For example, Yang and colleagues [33] developed an immunosensor for the detection of *Salmonella* spp. exploiting the immobilization of gold nanoparticles onto a glassy carbon and have demonstrated improved performances in terms of sensitivity and stability. On the other hand, Wang [34] proposed the use of TiO_2_ nanowires connected by gold microelectrodes and functionalized with anti-*Listeria* antibodies for the rapid and sensitive detection of *Listeria monocytogenes*. In this case, both a high sensitivity and a high specificity in combination with a fast response time were reported.

Recently, advances in microfabrication and microfluidics and the development of lab-on-a-chip technology (LOC) has offered new possibilities in many fields and also in microbiology and food security monitoring. In fact, LOC is the most suitable approach to address the challenge of bacterial isolation, thanks to the higher surface-to-volume ratio in a microchannel (which increases the active area of a microdevice for cell capturing) and the possibility to integrate other components, such as micropillars, micropores, microfilters and mixers, enhancing capture efficiency. Therefore, the future will be based on integrated platforms in which biosensors will be integrated with additional modules in order to perform all of the analytical procedures on the same chip.

For example, Jiang [35] has moved towards such an integrated platform by presenting a low-cost, miniaturized and sensitive bacteria sensor based on electrical impedance spectroscopy coupled with a smartphone platform. The proposed platform is composed of interdigitated electrodes on a micro-hole silicon substrate and a microfluidic chamber based on a nano-porous filter paper, which is used to pre-concentrate bacteria in sample solutions, which lowered the detection limit to 10 bacterial cells/mL.

Recent advancements in electrochemical impedance spectroscopy (EIS) and in differential pulse voltammetry (DPV) have been applied to detect DNA and protein-protein interactions on arrays [36].

### 2.3. Other Direct Methods

Semiconductor technologies have been also applied successfully in biosensor arrays. In particular, field-effect transistor technologies can play an important role: thin film transistors (TFTs) can be applied either as potentiometric sensors for the detection of different biomolecular interactions or as circuit elements for the conditioning and read-out of signals of promising label-free electrical detection techniques [37]. The use of nanostructured devices in chemical/biological sensors in place of conventional sensing technologies has the advantages of high sensitivity, low decreased energy consumption and potentially highly miniaturized integration. In this respect, owing to their particular structure, excellent electrical properties and high chemical stability, carbon nanotube and graphene-based electrical devices have been widely developed for high performance label-free chemical/biological sensors [38].

Surface acoustic wave biosensors and quartz crystal microbalances (QCM) have been applied to detect *E. coli* O157 strains in diluted samples [18]. Functionalized god nanoparticles were added to enhance the change in detection signals. Pathogenic *E. coli* was detected in food specimens using an amperometric immunosensor based on a self-assembled gold surface with immobilized gold nanoparticles and chitosan-multiwalled carbon nanotubes-SiO_2_-thionine, in a layer-by-layer film deposition process. The electrode connected with an amperemeter allowed for the visualization of bacterial cells, ranging from 400 up to 10^5^ CFU/mL [39]. The capacitative immunosensors based on QCM were shown to detect *E. coli* cells with a detection limit of 100 CFU/mL in inoculated food samples, using an electrochemical detector [39]. The specific capture of viable pathogens was followed by capacitative detection on a quartz crystal microbalance sensor followed by detection with antibody-functionalized gold nanoparticles [2]. In a different quartz crystal-based setting, the change in capacitance caused by the bacteria was directly measured with good sensitivity [40].

On-chip surface-enhanced Raman spectroscopy (SERS) [41,42] has been applied to pathogen detection. Surface-enhanced Raman spectroscopy (SERS) is a Raman spectroscopic (RS) technique that provides a greatly enhanced Raman signal from Raman-active analyte molecules that have been adsorbed onto certain specially prepared metal surfaces. Multispectral technologies, such as Raman, have been applied to individuate, through spectral profiles, single compounds, as well as multi-analyte in complex matrixes [41,42]. Electromagnetic spectrum wavelengths useful for multispectral technologies fall below the range of 10^−8^ meters. SERS technology is both surface selective and highly sensitive. Using a hybrid electrokinetic mechanism, it is possible to concentrate bacteria at the stagnation area on the SERS-active roughened electrode, easily identifying different pathogens by their characteristic spectra. Since this method does not need capture antibodies to selectively concentrate the investigated pathogen on the surface, it is not an immunosensor. On-chip SERS is based on the electromagnetic effect due to the excitation of localized surface plasmons. The observed signal enhancement is a consequence of the specimen physically adsorbed onto the surface.

Fourier transformation-infrared spectrometry (FT-IR) was used in label-free IR fingerprinting [43]. A benchtop FT-IR spectrometer and a portable mid-IR spectrometer were used to characterize *Salmonella* spp. and *E. coli* O157 strains in complex food matrices, such as diluted milk and spinach extract, after immuno-purification using magnetic beads. 

Another biosensor system has been based on thermally-blocked magnetic nanoparticles. Magnetic nanoparticle detection systems are based on an AC susceptometer measuring a wide frequency range, from 1 Hz up to 500 kHz. The magnetic flux change is measured by a detection-coil system connected to the AC source, delivering the current to the excitation coil that gives the excitation field. Signals are fed into a low-noise lock-in amplifier. This biosensor can detect the concentration of different molecules in a liquid [44]. The software elaboration of the magnetic field measurements in Tesla units results in a graphical resolution of plots with the frequency on the *x-*axis and magnetic susceptibility on the *y*-axis. Recently, this method was applied to measuring the presence of *Mycobacterium tuberculosis* in sputum samples, with a sensitivity in the range of 100–1000 CFU/mL, to individuate the acute phase of tuberculosis and to avoid the spreading of infection to healthy individuals [45].

The recent development of molecular electronics also offers new opportunities for implementing miniaturized and ultrasensitive transducers. So far, these methods have not been applied to bacteria detection but no constrains exist in this respect. At the molecular level, sensitivity is huge and, in one study, a nanobiosensor able to detect even single hybridization events was demonstrated by using arrays of nanojunctions and oligonucleotides conjugated to gold nanoparticles [46]. In these devices, target-probe binding events on the electrodes result in a quantized change in conductivity as nanoparticles close the gap. More recently, a protein transistor was made of an antibody molecule and two nanoparticles connected with two electrodes [47]. By means of CdSe quantum dots attached to the antibody, it was shown that the protein transistor can be gated by an applied optical field in addition to the electric signal detection. The authors demonstrated a reproducible approach to create a stable molecule/electrode interface [48] and successively applied this technology for DNA sequencing [49].

Additional methods exploited in label-free biosensors have been described and discussed by other authors, such as optical ellipsometry (OE), oblique-incidence reflectivity difference (OI-RD), resonant cavity biosensors and resonant waveguide grating (RWG) [16]. Optical biosensors have also been studied with an integrated Mach-Zehnder interferometer for the detection of *L. monocytogenes* [50]. Reflectometric interference spectroscopy (RIfS) has been applied by ForteBio (Menlo Park, CA, USA) in their Octet system for the monitoring of several different target molecules at the same time [16]. 

However, most of these methods, as in other biosensor systems, require costly detectors, so that applications to food pathogen detection are limited. 

## 3. Label-Based Detection Methods

Biosensor devices for pathogen detection generally consist of several elements, including a biological capture molecule (affinity probes or antibodies), a labelled antibody interacting with the bacteria captured from the solution (in the case of beads, a double label is already present on each different bead type) and a signal detection system.

Fluorescent methods make use of a detection system that reads the fluorescence signal of excited molecules, such as cyanines and Alexa Fluor dyes, or CdSe/ZnS quantum dots (QDs); the QDs possess high stability over time, but are excited on average by 50% of the molecules present on the sensor. Other sources of fluorescence are in the red and infrared wavelengths. Examples of such dyes are the HiLyte dyes, with high brightness, photo-stability and intense fluorescence, which are insensitive to pH changes. Quantum dots have been used to visualize *Salmonella* spp. enriched from a sample using immunomagnetic separation [6]. QDs exhibited a fluorescence emission peak at 620 nm, with a red shift of the conjugates of QD-dBSA-biotin (QD-dBSA, 625 nm) and QD-Ab (630 nm).

Some biosensors can be automated; many methods also have a multiplex capability, and some have high-throughput performance. Examples of such methods include electrochemiluminescent (ECL) assays performed in 96-well plates and cytometry-based assays that use antibody-coated microspheres. ECL detection is based on electrochemical stimulation of reporter molecules, which undergo electron transfer reactions, resulting in the emission of light. These reporter molecules, such as ruthenium(II) trisbipyridal (Ru(bpy)_3_^2^^+^), are attached to biological conjugates (e.g., antibodies) and, when stimulated by an electrode, allow detection at very low concentrations [51]. Instrument-specific 96-well assay plates are available for use with fully-automated, commercially available ECL detection systems (MesoScale Diagnostics, Gaithersburg, MD, USA).

Cytometric bead arrays (CBAs) make use of a fluidic method in which microspheres, individually coded using different ratios of red and infrared fluorophores, serve as the solid support for a sandwich-type immunoassay (xMAP technology, Luminex Corp., Austin, TX, USA) [52]. Capture molecules are attached to the microspheres and bind the target, while an orange-fluorescent reporter molecule (R-phycoerythrin (R-PE)) is used to generate the signal. The reporter for immunoassays is an R-PE-tagged antibody or a biotinylated antibody used in combination with streptavidin-conjugated R-PE. The bead/target/reporter complexes are read by a specialized flow cytometer, which determines the identity of each individual bead (based on the combined red/infrared fluorescent molecules) and the target-associated signal on each bead (based on the orange fluorescence). Hundreds of different sets of coded beads can be distinguished, allowing detection of up to 100 targets in one sample. Superparamagnetic fluorescent microspheres can also be used to incorporate immunomagnetic separation (IMS) procedures [52].

Lateral flow immuno-separation is a well-established method exploited in many commercial kits for bacterial identification after enrichment broth culture. Several types of substrates, membranes and detection systems are available, such as colorimetric or fluorescent quantification. In lateral flow immuno-assays for the identification of *Salmonella* spp., the detection antibody was labelled with gold nanoparticles (AuNP) and detected by densitometry using UV wavelength [53]. In another protocol, mastitis bacteria were detected using biotinylated antibodies and streptavidin coupled to carbon nanotube labels [54] visualized by flatbed scanning of the pixel gray intensity. 

### 3.1. Protein Chips, Antibody Chips and Aptamer Chips

Planar protein arrays in microplates [55], antibodies bound to microarray glass slides [4] and middle-throughput wells in microplates have been combined with magnetic separation and fluorescent silica nanoparticles in solution [56] and applied to the detection of pathogens and their toxins in the immunoassay format. 

The protein chip methodology is based on the binding to a glass surface of an antibody or an aptamer recognizing the bacterial species, for the capture of the bacteria to the surface. In our experiments (unpublished data), we tested either capture aptamers bound to a streptavidin-coated surface or capture antibodies directly bound to the epoxy-modified glass slides. Since the bacterial wall harbors several protein antigens in different locations in the membrane, it could be possible to use the same antibody either as a capture antibody or also as a detection probe. In the protein chip experiment, an activated surface, such as clean glass treated with a suitable chemistry, such as epoxy-groups, for the binding of amino residues (by a Schiff reaction and a reduction step to stabilize the Schiff base), is spotted with capture antibodies, streptavidin or proteins with high affinity for a bacterial species. The capture step can be either species-specific or genus-specific, if the detecting antibody shows high specificity for *L. monocytogenes* and does not bind to *Listeria innocua*. 

Detection antibodies can be directly labelled or detected using specific proteins (protein A, anti-IgG antibodies) that have been efficiently labelled. The fluorophores used more for such applications are Cyanine3/Alexa Fluor 555 with excitation/emission values at 550/568 nm, and Cyanine5/Alexa Fluor 647, with excitation at 650 nm and emission at 668 nm. Furthermore, species-specific biotinylated aptamers can be used for the detection step, combined with fluorescence-labelled streptavidin.

The detection method used can differ depending on whether the assay is in solution or on a surface, making use of laser scanners, cytofluorometer, fluoro-imagers or fluorescence microplate readers [4,57]. However, a portable and simple detection system is not yet available. Yet, improvements have been made to increase the sensitivity of fluorescence detection using a low-cost CMOS webcam and dedicated software readers [56]. The authors reported that by using a multi-wavelength LED illuminator and suitable filters, the photo camera acquired a video that was processed for signal-to-noise reduction by the software, achieving a limit-of-detection of 30 μM fluorescein in independent spots. In another report, a portable microarray slide reader measuring 19 cm in length has been developed that performs as well as commercial laser scanners, but that is much cheaper [58].

#### Approaches for Enhancement of Signal

A further approach to obtain an increase in sensitivity is to exploit multiple fluorescent labelling of the molecular recognition elements that bind to target bacteria. Using an antibody chip with spotted *Salmonella* spp. cells in serial dilutions, the detection method of choice is to incubate the slide with fluorescently (Alexa or cyanine)-labelled anti-*Salmonella* antibodies. We found that the sensitivity of this protocol was approximately 10^4^ CFU/mL. It was shown that lactoferrin (Lf) and lactoferricin peptides exert antimicrobial activity through binding to bacterial membranes. Recently, the binding of fluorescent Lf to bacterial membranes was shown in *Salmonella* cells bound onto glass slides. The fluorescence of Lf binding was at levels similar to the results obtained with boro-dipyrromethene boron fluoride (BODIPY), a lipid probe with excitation/emission at 530–550 nm, and to the anti-*Salmonella* antibody [53]. These findings indicate the feasibility to increase the detection of *Salmonella* spp. and related species of bacteria on protein chips by additive fluorescence detection using, at the same time, the anti-*Salmonella* antibody, BODIPY and labelled lactoferrin. 

It is possible to exploit two different monoclonal antibodies recognizing two proximal epitopes on the same surface antigen. The antibodies should be provided with an oligonucleotide tag, to exploit the amplification of the signal using the proximity probe ligation assay (PLA) [59]. In this method, it is possible to detect even single molecules in different formats, from *in situ* hybridization to confocal microscopy to capture antibodies fixed on glass slides or microplates. These methods resemble the use of fluorescently-labelled nucleic acid dendrimers to amplify the hybridization signals of DNA sequences bound to oligonucleotide arrays.

### 3.2. Fluorescence Resonance Energy Transfer (FRET)

Real-time monitoring of ligand-receptor interactions was performed on bacteria bound to carbohydrates exposed onto liposome surfaces using fluorescence resonance energy transfer (FRET). Ligand-receptor interactions were investigated at the bilayer surface using electronic absorption spectroscopy and fluorescence resonance energy transfer [60]. Since fluorescence is intrinsically more sensitive than colorimetry, the detection limit of the assay is in the sub-nanomolar range or lower. Polydiacetylene (PDA) liposomes were made with Lissamine Rhodamine (LR)-tagged phospholipids inserted in the bilayer, where LR acted as an acceptor (maximum absorption ~560 nm and maximum emission ~583 nm). Further, polydiacetylene (PDA) acts as a universal acceptor in FRET, which means that multiple sensors can be developed with PDA (acceptor) functionalized with donors and different receptors attached on the surface of PDA liposomes. With the addition of *E. coli* to PDA solution, the blue absorption peak at 540 nm was decreased and the intensities of the red absorption peaks (centered at 490 and 540 nm) were increased. The FRET emission response, on the other hand, depends on the FRET efficiency and on the distance between the donor and the acceptor (mean optimum radius at 50 nm).

### 3.3. Total Internal Reflection Fluorescence (TIRF) 

Total internal reflection fluorescence (TIRF) microscopy has been used to observe the fluorescence of single molecules. The region visualized, a few hundred nanometers wide, allows the visualization of the specimen zone surrounding the evanescent wave under a coverslip layered with a drop of immersion oil, achieving a selective description of surfaces at a high axial resolution [61]. Fluorophore conjugates are excited by means of an evanescent field generated upon total reflection of laser light at the surface. The short penetration depth avoids the excitation of fluorophores distant from the surface.

## 4. Conclusions

Several different types of biosensors, based on direct detection and indirect methods, such as lateral flow immunoassays and protein chips, have been reviewed in food pathogens detection. The assay performance, including detection limit and assay time, were also compared. The problem of the portability of the instrumentation has been approached with the development of cheaper, small-sized scanners and with software for the analysis of video images using mobile devices. Each biosensor technique has its own advantages and disadvantages in terms of the equipment required, sensitivity, simplicity and cost-effectiveness. Lab-on-a-chip (LOC) devices have a strong potential to be used in the field, since they can be miniaturized and automated; also being potentially fast and very sensitive. There are still several issues to be solved before in-field applications, including the pre-treatment of a sample, such as the enrichment of bacteria in culture broth, the proper storage of reagents, the full integration into a battery-powered system, the detection limit and the enhancement of the sensitivity of each method.
